# Tympanic Paragangliomas: case reports

**DOI:** 10.1016/S1808-8694(15)31293-3

**Published:** 2015-10-20

**Authors:** Maria Eugênia L. R. B de V. Neto, Isabela M. de Vuono, Luiz R.O. Souza, José R.G. Testa, Gilberto U. Pizarro, Fernando Barros

**Affiliations:** 1 Resident Physician, Hospital Paulista de Otorrinolaringologia; 2 Ph.D., Professor, UNIFESP-EPM; 3 Post-graduate studies under course, Discipline of Otorhinolaryngology, Escola Paulista de Medicina; 4 Otorhinolaryngologist

**Keywords:** glomus tympanicum, paraganglioma, glomus tumor

## Abstract

Glomus tumors, also called paragangliomas, originate from nonchromaffin cells. The tumor is typically vascular and grows from capillary and pre-capillary vessels in-between epithelial cells. It is worth mentioning that the most common symptoms are pulsating tinnitus and hearing loss. Imaging studies (CT and MRI) are necessary for diagnosis. This paper shows five patients seen at the Hospital between 1995 and 2001 presenting glomus tympanicum. Women were most commonly affected, and the age ranged from 48 to 60 years (mean age of 50 years). The most common complaints were pulsating tinnitus and hearing loss. All patients were treated surgically.

## INTRODUCTION

Glomus tumor affecting the temporal bone is an uncommon neoplasia of the middle ear. These tumors grow from paraganglion or glomus bodies, normal structures of the temporal bone. Glomus bodies originate from nonchromaffin cells; these cells, originating from the primitive neural crest^1^, compose the extra-adrenal neuroendocrine system. They may be found in the carotid body, adrenal medulla, and roof of the jugular bulb, along Jacobson and Arnold's nerves, from the jugular fossa to the promontory of the middle ear. Therefore, glomus tumors must be named according to their origin: glomus tympanicum, glomus jugularis, glomus vagale, glomus carotid^2^. According to Gaffey, paraganglions have chemoreceptor properties, and are named chemodectoma; however, jugulotympanic paragangliomas rarely cause blood pressure changes, although amine-secreting granules were found inside glomus cells. This may cause increased catecholamine synthesis^3^.

According to Rosenwaser, histologically, the material from the middle ear contained in tumor cells has granular eosinophilic cytoplasm and small oval nucleus, separated by fibrous tissue containing dilated veins. Tumor cells are close to capillaries, and sometimes they seem to project into the lumen. It is worth mentioning that intravagal tumors have less vascularization, in comparison with jugulotympanic paragangliomas. Polymorphism and hyperchromatism are frequent; however, these features are not associated with malignancy^1^, ^4^.

The most frequent symptoms are hearing loss and pulsating tinnitus, usually unilateral; adjacent structures are usually eroded due to massive vascularization. Tumor growth into the middle ear can destroy the ossicle chain, resulting in conductive hearing loss, facial palsy when fallopian tube is affected, and projection into the outer ear. Otoscopy shows a purple-red mass in the middle ear. Larger masses may be noted as polyps in the external ear and they may present massive bleeding when handled^5^, ^6^.

**Figure 1:**
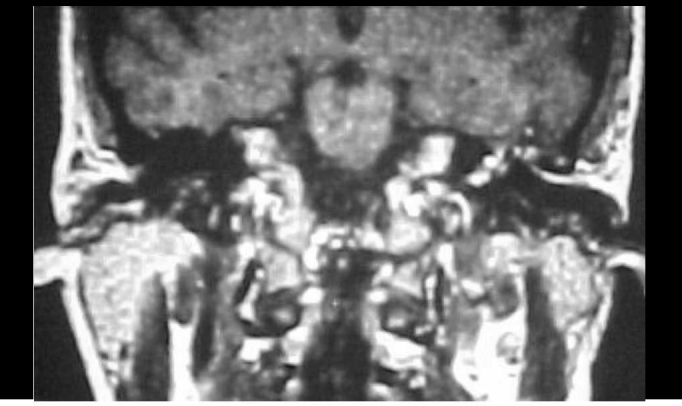
MRI of temporal bone using gadolinium contrast showing hypervascularized tissue in the left middle ear.

Audiometry shows either conductive hearing loss during early development, or sensorineural hearing loss, whenever the cochlea in affected. Impedanciometry may help the diagnosis: during the examination, tumor pulsation will shift the balance-meter needle synchronically to patient's pulse^1^. Diagnosis is confirmed through imaging studies. Biopsies are unnecessary and contraindicated. The best image study is CT scans, which shows tumor growth. The most common bone changes include widening of the jugular foramen and loss of bone lining (jugulotympanic paragangliomas) and soft tissues debris filling the ear opening in tympanic and jugulotympanic tumors, affecting the middle ear. MRI studies are recommended whenever there is evidence of intracranial invasion, as well as neck and large vessels involvement. Another possible diagnostic study is digital angiography, which will show features of vascular tumor and its vascular supply^7^.

The only treatment that can lead to cure is surgical removal. In patients where surgery is contraindicated, radiotherapy can be indicated. In tympanic tumors, surgery is carried out in the middle ear; an amplified transcanal approach may be enough to entirely remove the tumor. In cases where the tumor affects aditus and mastoid, a transmastoid approach and removal of the posterior part of the ear canal is indicated^5^, ^7^.

We will report 5 cases examined at the Hospital from 1995 to 2001.

## CASE REPORT

### Case 1

**Figure 2:**
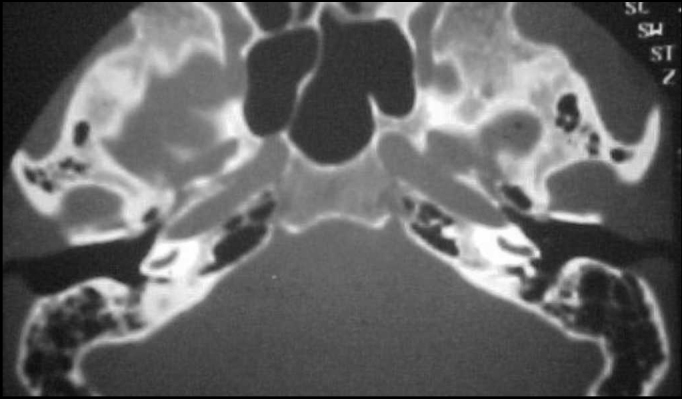
CT scan of temporal bone showing velamentum of the promontory and recess of the right facial nerve.

L. D., 49 years old, female, complaining of pulsating tinnitus on her right ear and ipsilateral hearing loss for 5 years. Otoscopy showed retrotympanic purple tumor; audiometry diagnosed mixed-type hearing loss, without discrimination, SRT of 80 dB. Tympanic mastoidectomy and resection of the glomus tumor was carried out, resulting in massive cavity. Histopathologic examination confirmed the diagnosis of glomus tumor. Postoperative audiometry showed mixed hearing loss and SRT of 90 dB. There was no recurrence after 3 years.

### Case 2

S. B. A., 50 years old, female, complaining of pulsating tinnitus and hearing loss on her right ear for four years. Otoscopy showed retrotympanic red tumor; audiometry diagnosed conductive hearing loss and gap of 25 dB. CT scan showed retrotympanic tumor. Tympanic mastoidectomy and tumor resection were carried out.

### Case 3

M. C. S., 53 years old, female, complaining of pulsating tinnitus for two years and hearing loss for one year on her left ear. Otoscopy showed retrotympanic tumor; audiometry diagnosed mild conductive hearing loss on her left ear. CT scan showed retrotympanic tumor. She underwent surgery through retroauricular approach, for complete removal of the tumor. Postoperative audiometry was normal.

### Case 4

I. M., 46 years old, female, complaining of pulsating tinnitus and hearing loss on her right ear for two years. Audiometry diagnosed mild sensorineural hearing loss of 15 dB. CT scan showed retrotympanic tumor and thickening of the right side of the mastoid. Tympanic mastoidectomy and tumor resection were carried out. Postoperative audiometry showed the same mild hearing loss of 15 dB.

### Case 5

T. C., 60 years old, female, complaining of frequent tinnitus and hearing loss on her right ear for one year. Otoscopy showed retrotympanic red tumor. Audiometry diagnosed mild conductive hearing loss. CT scan showed tumor on meso- and hypotympanic areas. She underwent surgery through retroauricular transcanal approach and total tumor resection. Postoperative audiometry showed sensorineural hearing loss in high frequencies. Control CT scan was normal.

## DISCUSSION

Glomus tumors are uncommon slow-growing and hypervascularized benign tumors. Gaffey called them chemodectomas, he believed that jugulotympanic paraganglions had chemoreceptor properties, and associated them with aortic and carotid bodies, which could modulate cardiopulmonary reflexes from changes in pO2 and blood pressure. They originate from glomus bodies, non-functioning chemoreceptors also located on the temporal bone, originating from the primitive neural crest. Clinically, the first symptoms of glomus tympanic are pulsating tinnitus and sensorial hearing loss; these symptoms were reported in all patients. The most frequent symptom reported in the literature is pulsating tinnitus, evidenced in all of our patients. However, due to the slow growing feature of this tumor, the symptoms will often occur when the tumor has already grown. The second most common symptom is hearing loss, conductive at first; when the inner ear is affected, in cases of central tumor growth, sensorineural hearing loss may occur. This occurred in our patients, possibly due to late diagnosis^8^.

**Figure 3:**
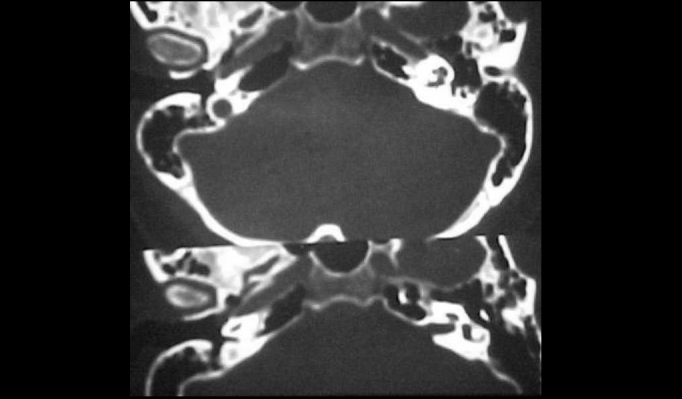
CT scan of temporal bone (axial section) showing circumscribed velamentum of the right promontory.

**Figure 4:**
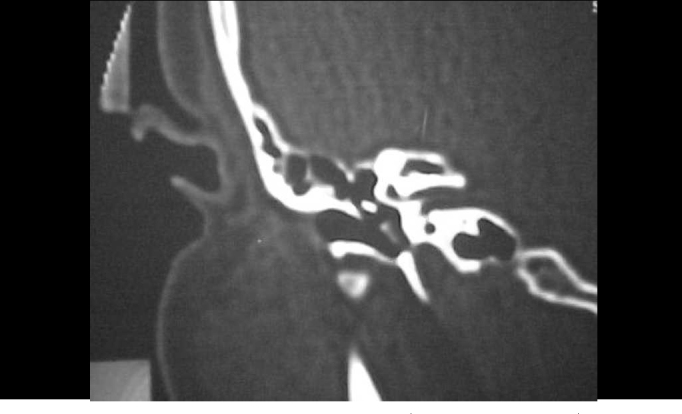
CT scan of temporal bone (coronal section) showing circumscribed velamentum of the right promontory and hypotympanum.

**Figure 5:**
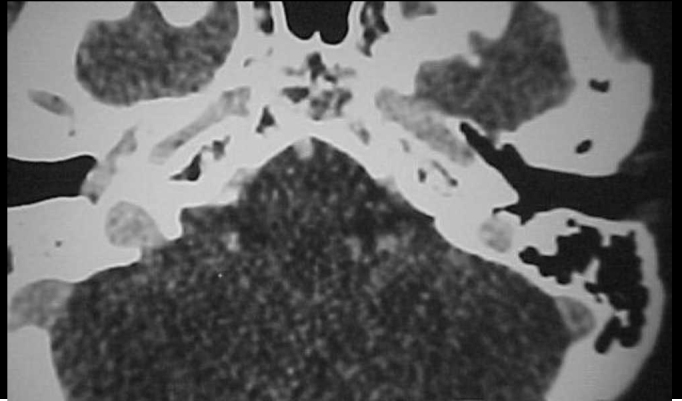
CT scan of temporal bone (axial section) with contrast showing velamentum of the middle ear towards the auditory tube.

The literature also mentions that females are more prone to developing this tumor, on a 4-6:1 rate: we corroborate this, however, we found a rate of 100%. The physical examination showed that the finding of purple retrotympanic mass during otoscopy is important; we could observe that in all cases. We chose to surgically remove the tumor in all cases through retroauricular approach, performing mastoidectomy whenever the tumor was too large. Tympanic mastoidectomy plus resection seemed to be associated with late diagnosis in our patients^4^, ^8^. The right ear was most commonly affected in all cases. This may be linked to the anatomy of the jugular gulf, more elevated and dilated on the right ear. However, we were unable to find data in the literature corroborating this incidence. As for transoperative period, it was always uneventful. All patients had complete or partial improvement in tinnitus.

Tympanic paragangliomas are slow-growing benign tumors in weak areas within the temporal bone. In the literature, there are rare reports of malignancy. The most common symptom is pulsating tinnitus, followed by conductive hearing loss^8^. The other symptoms are associated with tumor growth and bone invasion, facial nerve involvement through fallopian tube, infiltration on jugular foramen, auditory tube, carotid canal and sigmoid sinus^1^, ^2^.

As a result of these symptoms, audiometry and radiographs are mandatory (especially CT scan studies). Incision biopsies and paracentesis are contraindicated because there is high risk for complications resulting from bleeding. Surgical resection and histopathologic examinations are necessary to differentiate it from other tumors. When there is no contraindication, all patients must undergo surgery^3^, ^5^, ^7^.
